# Specific brain morphometric changes in spinal cord injury with and without neuropathic pain

**DOI:** 10.1016/j.nicl.2014.05.014

**Published:** 2014-06-02

**Authors:** Tom B. Mole, Kate MacIver, Vanessa Sluming, Gerard R. Ridgway, Turo J. Nurmikko

**Affiliations:** a Department of Psychiatry, University of Cambridge, Addenbrooke's Hospital, Level E4, Box 189, Hills Road, Cambridge CB2 2QQ, UK; b Unit of Neuroscience, School of Clinical Sciences, Pain Research Institute, Lower Lane, Liverpool L9 7AL, UK; c Department of Molecular and Cellular Physiology, Institute of Translational Medicine (PGR), University of Liverpool, Whelan Building, Liverpool L69 3GB, UK; d Wellcome Trust Centre for Neuroimaging, UCL Institute of Neurology, Queen Square, London WC1N 3BG, UK; e Walton Centre for Neurology and Neurosurgery NHS Trust, Lower Lane, Fazakerley, Liverpool L9 7JL, UK

**Keywords:** 10.020: Structural MRI, 40.100: Systems Pain, 40.130: Systems Somatosensory, 50.140: Pain syndromes Other, 50.160: Spinal cord, 50.180: Trauma, SCI, spinal cord injury, SCI-P, spinal cord injury with pain, SCI-N, spinal cord injury without pain, VBM, voxel-based morphometry, WM, white matter, GM, grey matter, ASIA, American Spinal Injury Association, BDI, Beck Depression Inventory, BAI, Beck Anxiety Inventory

## Abstract

Why only certain patients develop debilitating pain after spinal chord injury and whether structural brain changes are implicated remain unknown. The aim of this study was to determine if patients with chronic, neuropathic below-level pain have specific cerebral changes compared to those who remain pain-free. Voxel-based morphometry of high resolution, T1-weighted images was performed on three subject groups comprising patients with pain (SCI-P, *n* = 18), patients without pain (SCI-N, *n* = 12) and age- and sex-matched controls (*n* = 18). The SCI-P group was first compared directly with the SCI-N group and then subsequently with controls. Overall, grey and white matter changes dependent on the presence of pain were revealed. Significant changes were found within the somatosensory cortex and also in corticospinal tracts and visual-processing areas. When the SCI-P group was directly compared with the SCI-N group, reduced grey matter volume was found in the deafferented leg area of the somatosensory cortex bilaterally. This region negatively correlated with pain intensity. Relative to controls, grey matter in this paracentral primary sensory cortex was decreased in SCI-P but conversely increased in SCI-N. When compared with controls, discrepant corticospinal tract white matter reductions were found in SCI-P and in SCI-N. In the visual cortex, SCI-N showed increased grey matter, whilst the SCI-N showed reduced white matter. In conclusion, structural changes in SCI are related to the presence and degree of below-level pain and involve but are not limited to the sensorimotor cortices. Pain-related structural plasticity may hold clinical implications for the prevention and management of refractory neuropathic pain.

## Highlights

• Voxel-based morphometry was performed on spinal cord injury patients and controls.
• Patients with below-level neuropathic pain had reduced somatosensory cortex volume.
• Patients without pain had increased somatosensory cortex volume.
• Other structural changes were also found outside the sensorimotor cortices.
• Structural brain changes showed associations with the degree of neuropathic pain.

## 1 Introduction

Spinal cord injury (SCI) is a life-changing event that is known to cause characteristic cortical remodelling (Freund et al., 2013a; Freund et al., 2013b; Hou et al., 2014). However, it is unclear why approximately two-thirds of patients also develop unremitting neuropathic pain — and whether this brings specific underlying structural changes (Wrigley et al., 2009b).

Clinically, below-level neuropathic pain can be ‘excruciating’ (Siddall et al., 1997; Zatorre et al., 2012), overshadowed by motor deficits (Gustin et al., 2010) and challenging to treat, whilst strongly affecting quality of life (Boschen et al., 2003).

In the last decade, imaging studies in SCI have shown sensorimotor cortical atrophy and functional reorganization (Freund et al., 2011; Jurkiewicz et al., 2006; Wrigley et al., 2009a). Cortical areas representing deafferented leg regions in the sensory and motor cortices typically shrink and are invaded by expanding neighbouring regions (Freund et al., 2013a). Specifically, sensory cortex activation during brushing of a finger occurs medially towards the S1 region that would normally innervate the legs (Wrigley et al., 2009b). In addition plasticity in pain-processing regions as a result of pain has increasingly been recognized in various chronic conditions (Moseley, 2007; Price, 2002) suggesting that pain-related remodelling may additionally occur in SCI. Notably in deafferentation pain, the degree of S1 medial shift has recently been shown to correlate with on-going pain intensity (Wrigley et al., 2009b), potentially implicating the somatosensory cortex in developing neuropathic pain.

However, morphometry studies to date have been inconsistent (Crawley et al., 2004), outnumbered by functional studies, focused predominantly on grey matter (Freund et al., 2011) and importantly, have not differentiated patients with pain from those who are pain-free. We therefore aimed to determine whether pain-related morphological changes in SCI exist by disaggregating patient groups. We hypothesized that only SCI patients with below-level pain would have changes in pain-processing areas, including the somatosensory cortex.

## 2 Materials and methods

### 2.1 Study participants

A total of 48 subjects in three groups were recruited: 18 patients with below-level pain (SCI-P), 12 patients without pain (SCI-N) and 18 age-matched and sex-matched controls. Patients with SCI with all levels of impairment, as measured by the American Spinal Cord Injury Association (ASIA) scale A–D were recruited. This standardized functional impairment scale was used on all patients to assess limb function, with a higher ASIA score representing a higher level of function. The ASIA scores include three subscales for motor, pinprick sensation and light-touch sensation. The inclusion criteria were (i) T5–C5 cord injury, (ii) no neurological/mental illness, no high-dose opiates or drug/alcohol misuse and (iii) no contradictions for MRI. All patients also completed the Beck Depression Inventory (BDI-II) and Beck Anxiety Inventory (BAI).

### 2.2 Classifying clinical pain

Patients were only allocated to the SCI-P group if they had stable below-level pain for more than a year and over a period of 1 week before the first scan scored their daily pain intensity at 4 or greater on the Numerical Rating Scale (NRS) out of a range of 0–10. Patients were allocated to the SCI-N group if they were free from below-level pain or pain of other origin. Patients with only mild pain scores from one to three did not qualify for either group and were excluded from the study. This validated visual analogue scale was completed daily for 1 week prior to the structural scan. Following scanning, subjects were enrolled in a linked study investigating the effects of imagery techniques for chronic neuropathic pain relief.

### 2.3 Standard protocol approval, registration, and patient consent

All subjects gave written informed consent before participating in the study as approved by the South Central Local Research Committee in accordance with the Declaration of Helsinki.

### 2.4 MR-image acquisition

Patients and controls underwent the same imaging protocol with whole-brain MP-RAGE T1-weighted images on a 3 T Siemens Trio scanner. The imaging parameters were as follows: isotropic 1 mm^3^ resolution, field of view 256 × 256 × 192 mm with sagittal partitions, water excitation, repetition time 2300 ms, echo time 5.58 ms and flip angle of 8°. Images were checked for artefact and preprocessed with Statistical Parametric Mapping (SPM) 8 (http://www.fil.ion.ucl.ac.uk/spm) and the VBM8 Toolbox. First, 3D images were segmented into grey matter, white matter and CSF. Overall tissue volumes for grey matter, white matter and CSF were calculated using segmented images and expressed as fractions of their sum (the total intracranial volume). To achieve optimal inter-subject alignment, diffeomorphic non-linear image registration tool (DARTEL) was employed (Ashburner, 2007). Images were spatially normalized to Montreal Neurological Institute space, modulated for nonlinear change only and smoothed using an 8 mm full width at half-maximum Gaussian kernel. Processed images were analysed in SPM using the general linear model and random field theory. As previously employed, whole-brain analyses providing data-led findings were combined with hypothesis-led searches of a priori regions for improved sensitivity (Freund et al., 2011).

### 2.5 Whole-brain analysis

One-way analyses of covariance (ANCOVA) were performed to give statistical parametric maps showing regions of significantly different volume between groups. Age, total intracranial volume and sex were included as nuisance covariates to address potential confounds (Barnes et al., 2010). Volumes for significant clusters were extracted and compared across all three groups. Results are presented at *p* < 0.001 uncorrected for multiple comparisons. Clusters with peak heights that further survived family-wise error correction for multiple comparisons were also documented.

### 2.6 *A priori* regions

A priori regions were assessed by centring a 10 mm sphere on the following specific MNI coordinates: Primary motor cortex leg area (x = -6, y = -28, z = 60) (from Ciccarelli et al. 2005 in Freund et al., 2011), primary sensory cortex (S1) leg area (x = -4, y = -46, z = 62) (Ferreti et al. 2003 in Freund et al., 2011), left thalamus (x = -10, y = -20, z = 13) and right thalamus (x = 14, y = -24, z = 11) (Draganski et al., 2006). The left posterior cingulate (x = -16.91, y = -25.36, z = 40.01) and right insula (x = 38.7, y = 17.8, z = 7.22) coordinates were used from Preißler et al. (2013) after a validated Lancaster Talairach to MNI space transform was applied. White-matter coordinates for the left and right pyramids, posterior limb of the internal capsule and insula were extracted from the ICBM-DTI-81 atlas, as done previously (Freund et al., 2011). Significance for a priori regions was set at *p* = 0.05, corrected for multiple comparisons using family-wise error at the peak-level (with small volume correction).

### 2.7 Correlation analysis

Regions of interest were assessed for correlations with pain scores and six other clinical parameters: time since injury, ASIA-L, ASIA-M, ASIA-P, Beck Depression Inventory and Beck Anxiety Inventory. SCI-N and SCI-P groups were analysed separately. In particular, sensory impairment variables were assessed. Tissue volumes were extracted for significant clusters found on whole-brain and a priori analyses using the MARSBAR toolbox (Brett et al., 2002) and transferred into Statistical Package for the Social Sciences (SPSS) 20 for analysis using two-tailed Pearson's correlations.

## 3 Results

### 3.1 Clinical data

Thirty patients with thoracic and cervical SCI were recruited: 18 patients with pain and 12 patients without pain (see Table 1 for subject demographics and characteristics).

Comparisons across the three subject groups showed no significant differences in age (ANOVA, *f* = 2.22 *p* = 0.120) or in sex (chi-square, *p* = 0.086). When comparing the SCI-P and SCI-N groups, depression scores were significantly higher in the SCI-P group (12.39) compared with those in the SCI-N group (3.91) (independent *t* test *t* = 2.9 *p* = 0.006). Anxiety scores were also higher though this did not reach statistical significance (*t* = 1.7 *p* = 0.052). The mean (SD) time since injury was not significantly different in pain (11.1 [8.74]) and no-pain groups (17.7 [11.90]) (*t* = -1.74 *p* = 0.094). Likewise, clinical characteristics in the SCI-P and SCI-N groups including ASIA-M, ASIA-L and ASIA-P subscores and level of injury were not significantly different.

### 3.2 Whole-brain tissue fractions

No significant changes in grey matter, white matter or CSF fractions were found between SCI-P and SCI-N groups and controls (see Supplementary data in Appendix A.1). An increased CSF volume relative to total intracranial volume was found when all SCI patients were aggregated (0.188) versus controls (0.175) (*df* = 46 *t* = 2.012 *p* = 0.05).

### 3.3 Regional group differences

Overall, both grey and white matter changes were found in three principal areas comprising the deafferented S1 leg area, corticospinal tracts and visual-processing areas (Table 2). Comparisons of SCI-P and SCI-N aggregated together versus controls can be found in the Supplementary data (Appendix A.2).

Only one a priori region of interest, in S1, showed a significant difference. SCI-N had significantly increased S1 grey matter relative to controls in the left hemisphere (*p* = 0.048 FWE-corrected). Increased S1 grey matter was also found in the right hemisphere but this failed to reach significance (*p* = 0.101 FWE-corrected).

**Fig 1:**
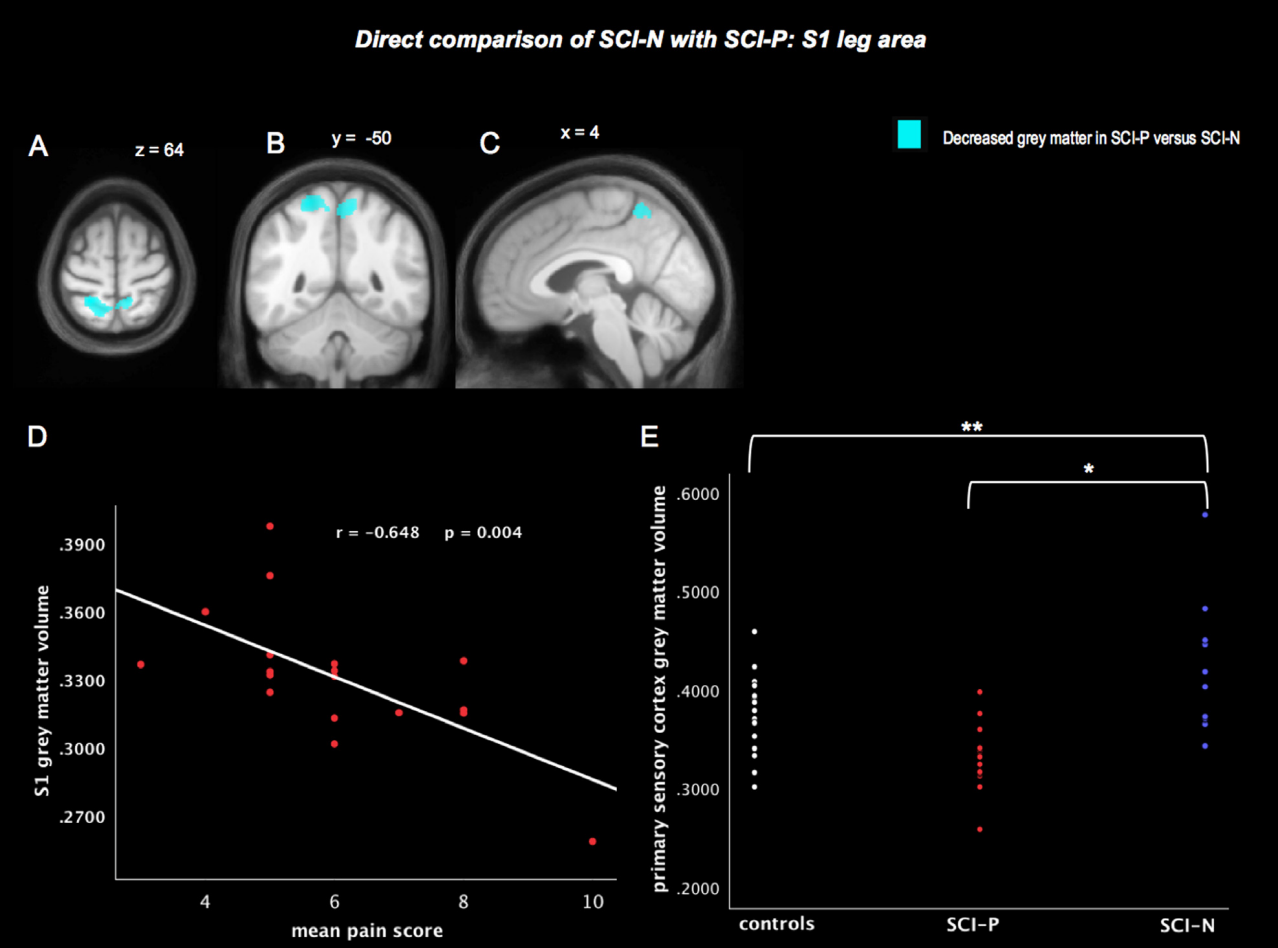
Direct focal volume comparison of SCI-N with SCI-P: S1 leg area. Averaged S1 volumes were extracted from the supra-threshold cluster formed at a threshold of *p* < 0.001 from the whole brain SCI-P versus SCI-N contrast. Divergent changes in S1 (grey matter) were found in SCI-P and SCI-N groups compared to healthy controls. The SCI-P subgroup had significantly decreased grey matter in S1 leg area bilaterally (A–C) compared with the SCI-N subgroup (E*). Focal grey matter volumes for this S1 region correlated negatively to pain scores in the SCI-P group (D). Focal grey matter volumes for the illustrated bilateral S1 cluster varied across the three study groups (E). In whole-brain analyses, relative to controls, there was no significant increase in S1 grey matter in the SCI-N group (E**). Notably, however, from the a priori S1 region of interest with its increased sensitivity, a statistically significant increase was found in the left S1 leg area in the SCI-N group (*p* = 0.048) and also an increase in the right S1, though this did not reach significance (*p* = 0.101). Image shows the cluster formed at a threshold of *p* < 0.001 (uncorrected).

In whole-brain analyses, the SCI-P group showed reduced grey matter in S1 bilaterally compared with the SCI-N group. A single cluster extended into both left and right hemispheres, most prominently in the left hemisphere, in areas that typically represent sensation from deafferented lower limbs (Fig. 1A–C). This area also was associated with pain scores (*r* = -0.648 *p* = 0.004) (Fig. 1D). To establish the relationship of paracentral S1 changes relative to controls, grey matter volumes for this cluster were extracted for all subjects. This showed a relative increase of mean grey matter in SCI-N and mean decrease in SCI-P though neither of these differences reached significance with whole-brain contrasts (Fig. 1E). Other than S1 changes, no further differences were found when SCI-P was compared with SCI-N.

**Fig 2:**
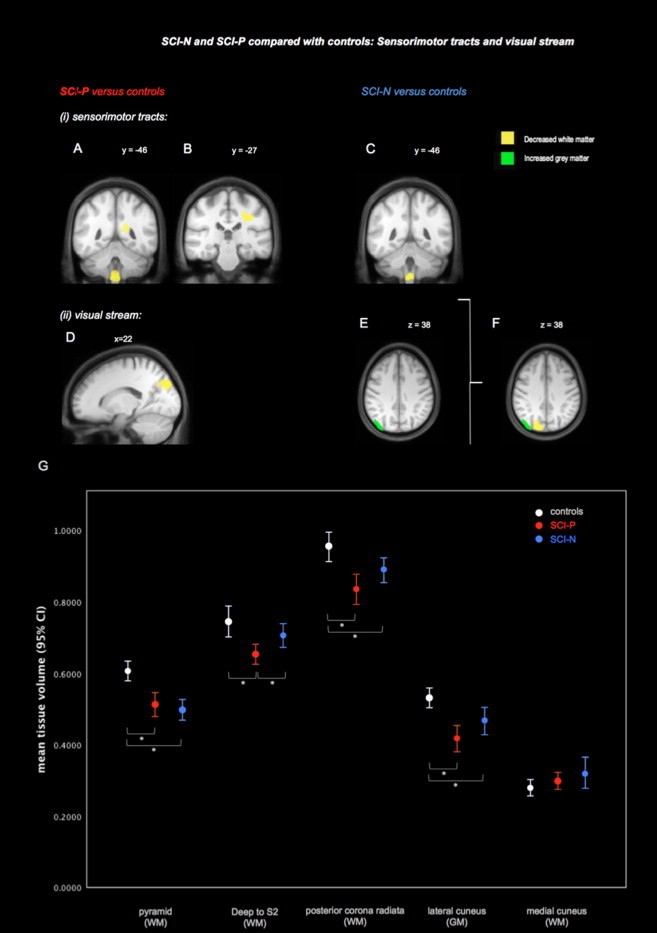
SCI-N and SCI-P groups compared with controls: averaged ROI volumes were extracted for the supra-threshold clusters identified from whole brain contrasts (*p* < 0.001 uncorrected) in Table 2. Changes were found in (i) the sensorimotor tract and (ii) visual processing areas. Areas associated with the corticospinal tract showed reduced volume (white matter). Compared with controls, the SCI-P group showed reduced white matter (WM) volume in the pyramids and also in two further regions: deep to S2 (A), and in the area of the posterior corona radiata (B). The SCI-N group showed white matter changes also in the pyramids but not in any other locations (C) (ii). Mixed visual stream changes were found (both grey matter and white matter). The SCI-P group showed a decrease of white matter medially in the cuneus (D). The SCI-N subgroup showed increased grey matter in the adjacent lateral cuneus extending into the precuneus (E). The anatomical relationship between visual cortex clusters is shown in (F). When tissue volumes were extracted for each region and compared across the three groups, variation between groups was revealed (G). Note that error bars indicate 95% confidence intervals for the mean and are based on raw extracted tissue volumes. Asterisks represent *t*-tests that showed significant group differences of averaged extracted supra-threshold cluster volumes (*p* < 0.05). All images are displayed on an averaged template based on all study participants, thresholded at *p* < 0.001 uncorrected.

When SCI-P and SCI-N groups were compared with controls, changes were found in the corticospinal tract and visual-processing areas (Table 1). Along the corticospinal tract, reduced white matter was found in both SCI-P and SCI-N groups in the pyramids bilaterally. For SCI-P only, two further white matter changes were found in the corticospinal tract deep to the left S2 and in the region of the right posterior corona radiata. In the occipital cortex, a further large cluster of reduced white matter was found only in the SCI-P group. This cluster was adjacent to the left occipital–parietal fissure and not only involved mainly the medial cuneus but also extended into the adjacent precuneus. In the SCI-N group, a grey matter increase was detected neighbouring the white matter change previously seen in the SCI-P group in the lateral cuneus (Fig. 2F). In contrast, the SCI-P group showed no grey matter expansion in the visual cortex.

### 3.4 Correlation analysis

In the SCI-P group, pain scores were negatively correlated with grey matter volume in the paracentral S1 cluster. Pain also correlated positively with white matter volume associated with the corticospinal tract deep to the secondary sensory cortex (S2) (*r* = 0.476 *p* = 0.046). This region further correlated with anxiety scores (*r* = 0.595 *p* = 0.009). Depression scores showed no associations with any region. All three measures of motor and sensory function (ASIA-L, ASIA-M, ASIA-P) and time since injury were correlated with pyramidal white matter volume. In the SCI-N group, one association was found, which showed cuneal white matter volume was negatively correlated with light touch sensory function (reduced ASIA-L) (*r* = -0.600 *p* = 0.039).

## 4 Discussion

We found that both SCI-P and SCI-N groups not only had common structural brain changes in the pyramids but also had divergent changes elsewhere — in the sensory cortex and areas not commonly associated with pain processing. Principally, we found changes in S1 that correlated both the presence and severity of pain (*r* = -0.648 *p* = 0.004) and novel evidence that S1 may have increased rather than decreased volume specifically in SCI-N. Further discrepancies in findings between SCI-N and SCI-P groups were found in the corticospinal tract and visual-processing areas, suggesting that these areas may also play a role in the development of deafferentation pain.

### 4.1 S1

There have been few studies exploring the integrity of S1 after either spinal cord or peripheral nerve injury. Typically, primary motor cortex changes from retrograde degeneration have been focused on. Evidence of changes in S1 after SCI is limited but experimental and clinical studies have shown that substantial reorganization occurs and that there may also be shrinkage of the sensory cortex representing deafferented areas (Freund et al., 2013b; Freund et al., 2011; Henderson et al., 2011; Hou et al., 2014; Jones, 2000).

Our finding of decreased grey matter in the paracentral area in SCI-P compared with SCI-N is in line with previous evidence showing the importance of S1 to below-level pain (Gustin et al., 2012). Mean grey matter volumes were also decreased in the pain group relative to controls, as previously found in SCI studies (Jurkiewicz et al., 2006), though this did not reach significance in our study. Overall, based on comparisons of grey matter volumes for S1 across the three groups, there may be an increase in grey matter in SCI-N — a novel finding, and a decrease in SCI-P relative to controls, although this potential relationship needs to be viewed with caution and confirmed with future studies. Decreased S1 volume may be due to reduced afferent input and may be associated with increased invasion of neighbouring cortex and increased functional reorganization (Henderson et al., 2011). On a cellular level, S1 volume reduction could reflect apoptosis, cell shrinkage and retraction of thalamocortical connections (Jones, 2000). Histopathological studies in monkeys in which deafferentation was induced by amputation or extensive rhizotomy show that the VPL nucleus of the thalamus undergoes atrophic changes following deafferentation and it is inferred that transneuronal degeneration similarly explains changes in the somatosensory cortex, especially when the deafferentation is longstanding (Jones, 2000). In addition, abnormal thalamic activity has been recorded from outside the atrophied regions of VPL (Lenz et al., 1994; May, 2008). Importantly, it has been shown that nervous system lesions can induce dysrhythmic thalamocortical oscillations within the thalamocorticothalamic circuitry that is considered to contribute to central pain (Stern et al., 2006). We suggest that the cortical counterpart of the loop is essential in the maintenance of this maladaptive activity, with reduction in S1 paracentral grey matter volume favouring development of pain and increase in volume protecting from it.

Increase in cortical volume in painful conditions and following injury is reported in imaging studies and is attributed to axon sprouting, dendritic branching, and neurogenesis, as well as angiogenesis and changes in glial morphology (Zatorre et al., 2012). Henderson et al. pointed out that the large-scale SI reorganization they witnessed in their spinal cord injury patients is best explained by lateral dendrite growth, rather than unmasking of silent synapses (Jurkiewicz et al., 2006). Intriguingly, some recent discoveries go even further suggesting that actual cortical neurogenesis may occur, with formation of new neurons in non-human primate S1 reported after spinal dorsal root injury (Vessal and Darian-Smith, 2010). Moreover, the majority (80%) of newly formed neurons were found in S1 with only a small number being found in the primary motor cortex which is in line with findings of substantial motor cortex atrophy found in SCI (Freund et al., 2011). Positive, compensatory brain S1 remodelling has previously been associated with skills training such as Braille (Freund et al., 2013a; Pascual-Leone et al., 1993). These findings support evidence that S1 remodelling could potentially be associated with both adaptive and maladaptive responses to pain. If the finding of increased grey matter is corroborated in future studies, the possibility of genuine neurogenesis and its role in pain prevention may warrant further consideration. Specifically, exploring local trophic mechanisms and neurochemistry that might affect dendrite growth could be pursued.

### 4.2 Sensorimotor tracts

Our findings support recent evidence from neuroimaging (Freund et al., 2011) indicating that the descending corticospinal as well as corticopontine (Wrigley et al., 2009a) tracts undergo significant changes following SCI. Given the variability in imaging studies to date, the repeated finding of pyramidal atrophy and strong correlations to a range of sensory, motor and clinical parameters suggest its potential as an imaging biomarker for SCI if confirmed in future studies. It is possible that white matter tract changes may be affected by pain as the current study showed discrepant findings in the SCI-P and SCI-N groups. Reasons why white matter may show changes related to pain are not clear. However, only in the SCI-P group were areas of white matter atrophy outside the pyramids in the corona radiata found, which were absent in the SCI-N group. Moreover, pyramidal correlations to clinical variables were exclusively found in the SCI-P group. This might suggest that patients with pain have more significant disruption to their white matter tracts. Pain-related white matter changes could be due to a range of factors including associations with lesion severity or motor disuse. One study (Werhagen et al., 2004) but not others (Siddall et al., 2003; Siddall et al., 1999) found that below-level pain was the only type of neuropathic pain that correlated with completeness of injury. Moreover, lesions leaving more profound motor impairment have also recently been correlated with greater white matter loss in the cerebral peduncles, but it is unclear if pain could have contributed to this atrophy as pain scores were not included in this recent study (Hou et al., 2014). Significant atrophic changes in spinal cord injury appear to occur in the early phase of the disease as this recent study showed prominent changes approximately 9 weeks after injury. On a cellular level, observed white matter loss is known to be driven by atrophy from retrograde degeneration, though this could also be due to contributions from processes such as reduced connectivity and reduced angiogenesis (Freund et al., 2013a; Freund et al., 2011; Zatorre et al., 2012).

### 4.3 Visual processing areas

We found discrepant plasticity within the visual cortex depending on the presence of pain. This is consistent with preliminary evidence linking changes in the visual-processing and pain such as the use of mirror boxes and visual imagery to modulate neuropathic pain (Chan et al., 2007; Moseley, 2007). In deafferentation pain from phantom limb pain (Preißler et al., 2013), precuneal grey matter increase was also associated with reduced pain, suggesting a potential adaptive mechanism in the visual stream in response to compensate for lack of sensory input.

Discrepant grey and white matter morphometric changes were found in the cuneus and adjacent precuneus, according to the presence of pain. One proposed role of the posterior parietal cortex is appraising pain before further second-order processing in the insula and anterior cingulate cortex (Price, 2002). Together with the cuneus, the precuneus also has a role in the processing of visual information in the ‘visual stream’ which is critical for spatial orientation and movement detection (Rossetti et al., 2003). Changes in visual-processing areas, including the precuneus, have recently been reported in a phantom limb pain study (Preißler et al., 2013), where deafferentation and chronic neuropathic pain coexist as found in spinal cord injury. Precuneal grey matter increases were similarly found in phantom limb pain patients compared with controls. It was found in this study that these visual-processing regions were associated with reduced pain, suggesting that occipital grey matter growth may represent a potential protective, adaptive mechanism against developing neuropathic pain. This notion is in line with evidence that visual feedback via mirror boxes can reduce deafferentation pain (Chan et al., 2007). Likewise, a clinically important association between the visual cortex and pain is further supported by evidence that visual illusions and ‘virtual walking’ may significantly modulate neuropathic pain (Moseley, 2007; Gustin et al., 2008; Soler et al., 2010). Consistent with these findings, our results of plasticity in the visual stream suggest that patients may have some form of visual changes to compensate for lack of sensory feedback from deafferented body parts. The lack of correlation between white matter volume reduction and intensity of pain suggests that rather than having a modulatory role, these changes reflect the propensity for developing deafferentation pain after spinal cord injury.

### 4.4 Limitations

The SCI-N group (*n* = 12) was relatively small compared with the SCI-P group (*n* = 18). Nonetheless, the study involved more SCI patients than previous structural studies. In addition, it is uncertain in the current study whether the differences observed when comparing the subgroups with and without pain to controls are due to insufficient study power or inherent group differences. Given that our findings show pain-related differences between patients, we recommend that future studies document patients' pain intensities so that plasticity contributions from pain and deafferentation can be further disambiguated. Together with longitudinal studies, dynamic changes associated with pain and its treatment could be determined.

## 5 Conclusion

In conclusion, despite several studies showing morphometric changes in SCI, no studies have compared pain-free patients to those with pain. We were able to show specific S1 changes in the deafferented leg area and discrepant findings in the sensorimotor tracts and the visual stream associated with the presence of pain. With the majority of neuropathic pain being resistant to conventional treatments, future work manipulating reorganization of the somatosensory (Wrigley et al., 2009a) and visual cortices may provide a different potential treatment approach. Such efforts at developing alternative approaches will be important if efficacious treatments are to be developed and quality of life improved for patients suffering from SCI and below-level pain.

## Disclosures

There was no corporate sponsorship of this study. The authors disclose no other financial relationships deemed relevant to the manuscript.

Dr Mole interpreted and analysed the data and wrote the manuscript. Dr MacIver designed the study, recruited patients and drafted the manuscript. Dr Sluming and Dr Ridgway analysed the data and drafted the manuscript. Prof. Nurmikko conceived the research idea, designed the study and wrote the manuscript.

Supported by MRC grant number MR/J014257/1, Wellcome Trust Centre for Neuroimaging grant number 091593/Z/10/Z, Inspire UK and a grant from the Pain Relief Foundation.

## Figures and Tables

**Table 1:** Clinical and functional characteristics for SCI subjects with and without pain.

Subject	Age (years)	Time since injury (years)	Motor impairment level	ASIA impairment score			Mean pain score week prior to scan	BDI	BAI
				Motor score	Light touch	Pinprick			
***SCI-P***									
1	52	5	C6	92	86	82	6	12	8
2	48	24	T1	50	31	36	5	21	6
3	69	17	C7	45	82	68	3	3	0
4	56	7	C5	82	8	25	5	9	3
5	59	24	T4	30	44	10	8	10	11
6	57	7	C6	95	95	78	5	13	21
7	45	2	T5	96	80	80	6	17	2
8	39	14	C5	44	59	103	8	15	6
9	49	25	C6	20	18	27	6	4	0
10	41	4	C5	93	96	82	4	28	3
11	53	5	T3	50	36	36	6	25	7
12	50	15	T5	44	61	60	10	2	0
13	46	5	C5	93	84	63	5	11	0
14	50	25	C5	10	18	18	5	2	1
15	46	14	T2	38	34	34	6	0	0
16	51	4	T1	50	42	42	5	4	0
17	68	1	C5	59	62	62	7	7	3
18	45	2	C6	95	102	105	8	40	18
Mean	51.3	11.1		60.3	57.7	56.2	6	12.39	4.9
Median	50.0	7.0		50.0	60.0	61.0	6	10.5	3.0
IQ range	10.5	14.75		50.5	51.25	48.25	2.25	14.25	7.25
***SCI-N***									
1	41	9	C5	74	112	112	0	1	0
2	65	17	C5	0	12	12	0	7	4
3	40	23	C5	32	25	27	0	2	0
4	71	17	C6	58	37	36	0	1	0
5	26	3	C5	62	104	104	0	5	5
6	71	23	T4	85	110	97	0	1	0
7	26	7	C5	64	44	50	0	6	3
8	49	31	T1	38	71	68	0	0	0
9	76	2	T4	50	43	41	0	17	5
10	61	40	C6	10	44	66	0	2	3
11	69	11	T5	50	38	28	0	5	0
12	56	29	T5	50	50	102	0	0	0
Mean	54.25	17.7		47.8	57.5	61.9		3.91	1.38
Median	58.5	17.0		50.0	44.0	58.0		2.0	0.0
IQ range	30.25	20.0		30.0	58.5	70.75		4.75	3.75

ASIA, American Spinal Injury Association; BDI, Beck Depression Index; BAI, Beck Anxiety Index.

**Table 2:** Significant group differences in grey and white matter.

	Region	Side	Volume change	Coordinates in MNI space			*p*-Value	
				x	y	z	Uncorrected	FWE-corrected
*Grey matter*								
SCI-P versus SCI-N	S1	L + R	Decrease	-22	-46	64	0.001	
SCI-P versus controls	Nil significant							
SCI-N versus controls	S1 (a priori region of interest)	L	Increase	-4	-46	61		0.048
	S1 (a priori region of interest)	R	Increase	4	-46	62		0.101
	Lateral cuneus	L	Increase	-33	-87	36	0.050	
*White matter*								
SCI-P versus SCI-N	Nil significant							
SCI-P versus controls	Pyramids	L + R	Decrease	-3	-37	-62	0.003	
	Medial cuneus (extending into precuneus)	L	Decrease	-15	-81	37		0.011
	White matter deep to S2	L	Decrease	34	-28	37	0.011	
	Posterior corona radiata	R	Decrease	18	-36	22	0.018	
SCI-N versus controls	Pyramids	L + R	Decrease	-4	43	-62	0.003	

## Appendix A Supplementary materials

Supplementary materials for Specific brain morphometric changes in spinal cord injury with and without neuropathic pain.
