# Supplementary material for: Specific brain morphometric changes in spinal cord injury with and without neuropathic pain
Source: Neuroimage Clin. 2014 Jun 2;5:28–35. doi: 10.1016/j.nicl.2014.05.014 (PMC4055864; doi:10.1016/j.nicl.2014.05.014)
Supplement: Supplementary file 1 — Supplementary materials for Specific brain morphometric changes in spinal cord injury with and without neuropathic pain. [file mmc1.docx]

**Appendix**

*A.1* *Whole-brain tissue fractions*

|  | Healthy controls | SCI-P |  |  | SCI-N |  |  | SCI-P & SCI-N |  |  |  |  |
| --- | --- | --- | --- | --- | --- | --- | --- | --- | --- | --- | --- | --- |
|  | (*n*=18)  Mean (SD) | (n=18)  Mean (SD) | T^a^ | p^a^ | (*n*=12)  Mean (SD) | T^a^ | p^a^ | (*n*=30)  Mean (SD) | T^a^ | p^a^ | T* | p* |
| GM | 0.427 (0.027) | 0.421 (0.025) | -0.705 | 0.485 | 0.412 (0.038) | -1.248 | 0.222 | 0.417 (0.031) | -1.097 | 0.278 | 0.749 | 0.460 |
| WM | 0.398 (0.024) | 0.391 (0.020) | -0.974 | 0.337 | 0.399 (0.023) | 0.132 | 0.896 | 0.394 (0.021) | -0.578 | 0.566 | -1.067 | 0.295 |
| CSF | 0.175 (0.019) | 0.188 (0.023) | 1.844 | 0.074 | 0.189 (0.025) | 1.643 | 0.112 | 0.188 (0.024) | 2.012 | 0.050 | -0.035 | 0.972 |

*A.2 Aggregated SCI-N and SCI-P groups versus controls: Gray and white matter differences*

|  | Region | Side | Volume change | Coordinates in  MNI space | | | p-value | |
| --- | --- | --- | --- | --- | --- | --- | --- | --- |
|  |  |  |  | x | y | z | uncorrected | FWE-corrected |
| *Gray Matter* |  |  |  |  |  |  |  |  |
| SCI-P + SCI-N combined versus controls | lateral cuneus | L | increase | -38 | -79 | 34 | 0.042 |  |
| *White Matter* |  |  |  |  |  |  |  |  |
| SCI-P + SCI-N combined versus controls | medial cuneus  (extending into precuneus) | L | decrease | -15 | -81 | 37 |  | 0.017 |
